# Germline *HLA* and *KIR* Variation and Disease Control After Immune Checkpoint Blockade in Non-Small Cell Lung Cancer: Exposure Definition Shapes the Signal

**DOI:** 10.3390/ijms27219933

**Published:** 2026-11-06

**Authors:** Andrea González-Hernández, Alberto Ríos, Juan Luis Onieva, Alexandra Cantero, Marina Rivero-Aguilar, Guillermo Paz-López, Antonio Rueda-Domínguez, María Garrido-Barros, Beatriz Martínez-Gálvez, Elisabeth Pérez-Ruiz, José Carlos Benítez, Isabel Barragán, Javier Oliver

**Affiliations:** 1 Facultad de Medicina, Universidad de Málaga (UMA), 29071 Málaga, Spain; 2 Servicio de Oncología Médica, Hospital Universitario Virgen de la Victoria, Grupo B-05, Instituto de Investigación Biomédica de Málaga-Plataforma BIONAND (IBIMA-Plataforma BIONAND), 29010 Málaga, Spain; 3 Servicio de Oncología Médica, Hospital Regional Universitario de Málaga, Grupo B-05, Instituto de Investigación Biomédica de Málaga-Plataforma BIONAND (IBIMA-Plataforma BIONAND), 29010 Málaga, Spain; 4 Estructura Común de Apoyo a la Investigación de Bioinformática, Instituto de Investigación Biomédica de Málaga-Plataforma BIONAND (IBIMA-Plataforma BIONAND), 29590 Málaga, Spain; 5 Precision Cancer Medicine in Lung Cancer–Preclinical, Translational & Clinical Research–Simon Ekman’s Research Group, Department of Oncology-Pathology, Karolinska Institutet, 171 64 Stockholm, Sweden

**Keywords:** non-small cell lung cancer, immune checkpoint inhibitors, HLA class I, HLA class II, killer cell immunoglobulin-like receptors, HLA supertypes, allele-group resolution, exome-based HLA typing, multiple testing, HLA evolutionary divergence

## Abstract

Immune checkpoint inhibitors (ICIs) benefit only a minority of patients with advanced non-small cell lung cancer (NSCLC). Whether germline human leukocyte antigen (HLA) or killer cell immunoglobulin-like receptor (KIR) variation identifies them is unclear, partly because studies define HLA exposures differently. We asked whether allele groups, supertypes and evolutionary divergence associate with disease control. In 122 ICI-treated patients, HLA and KIR typed from germline exomes were tested in 448 pre-specified exposure-endpoint combinations; the primary endpoint was progression or death at 3 months (99 evaluable patients; 31 events). No test survived correction across the screen (lowest *n* = 0.299). Nominally, HLA-A*02 carriers had higher odds of early failure (odds ratio 4.00, 95% CI 1.64 to 10.07), whereas none of the 12 HLA-B*14 carriers failed (0.07, 0.0006 to 0.58), yet the B27 supertype containing B*14 was null. In 297 public SU2C-MARK patients, B*14 was not supported; A*02 was inconclusive for time to event and not reproduced at a 90-day landmark. *HLA-DQA1* divergence at 12 months was hypothesis-generating (within-family *n* = 0.004). Exome typing resolved HLA allele groups (99.1%) but not KIR (19.0% ambiguous). How an HLA exposure is defined determines whether a signal is visible; only large effects were detectable; replication is required.

## 1. Introduction

Anti-PD-1 and anti-PD-L1 antibodies are standard first-line treatment for advanced non-small cell lung cancer (NSCLC) [1,2], but only a minority of patients achieve durable disease control [1,2,3]. PD-L1 expression is the established biomarker, yet its predictive performance is inconsistent across studies and assay platforms [2]. In an NSCLC discovery cohort with two validation cohorts, PD-L1 expression, tumour mutational burden and cancer gene mutations predicted benefit better than HLA class I genotype, which was not associated with survival [4]. Germline HLA nevertheless remains a plausible determinant: class I molecules present peptides to CD8 T cells [5], and germline HLA class II heterozygosity is associated with lower lung cancer risk in smokers, implicating antigen presentation in the control of early lung neoplasia [6]. Whether germline HLA matters for checkpoint response in NSCLC is unsettled.

Germline HLA has been examined at three levels of aggregation. Genotype-level summaries include class I homozygosity [7,8], HLA evolutionary divergence, the mean physicochemical distance between the two alleles’ peptide-binding domains [9,10], and composite scores of predicted neoantigen presentation across the genotype [11]; in checkpoint cohorts, divergence has been tested almost exclusively at class I [12,13,14]. Allele groups have been implicated in both directions, most prominently HLA-A*03 [15], and allele-level class I subtypes have also been reported to predict checkpoint response [16]. Supertypes, which pool alleles by shared peptide-binding motif [17], include a reported B27 association in advanced NSCLC treated with pembrolizumab plus chemotherapy [18] and an A02/B62 supertype associated with prognosis in stage I lung cancer [19]. KIR variation, which modulates natural killer cell licensing through HLA class I ligands [20,21], has been associated with resistance to PD-1 blockade in lung cancer [22] and with treatment response and survival in NSCLC [23].

Two gaps limit comparison between studies. Findings at different levels of aggregation are not interchangeable: a supertype pools alleles whose effects may differ, so an association reported for a supertype need not hold for its constituent alleles, nor an allele-group association for its supertype; and the same supertype can behave differently across settings, as B44 did when a melanoma association failed to reproduce in NSCLC except on stratification by somatic mutational landscape [24]. And studies tend to report the association found rather than the screen it came from, so readers cannot see how many exposures were tested or how reliably alleles were called.

Germline HLA and KIR genes could affect checkpoint response by shaping the tumour immune microenvironment. Class I molecules present peptides to CD8 T cells and class II molecules to CD4 T cells, so the HLA genotype determines which tumour peptides T cells can see [5,7,12]. In lung cancer, the tumour can also lose class I alleles: loss of heterozygosity at the HLA locus was found in 40% of NSCLC in the TRACERx cohort and was linked to a high subclonal neoantigen burden, cytolytic activity and PD-L1 positivity [25], and somatic HLA class I loss was associated with shorter overall survival after checkpoint blockade in nonsquamous NSCLC [26]. KIRs on natural killer (NK) cells recognise the HLA class I ligand groups C1, C2 and Bw4, and the receptor-ligand combination sets NK-cell responsiveness [20,21]; an NK-dendritic cell axis has been described in tumour microenvironments that respond to checkpoint therapy [27]. The germline genotype is fixed for life, can be read from blood or an existing exome and does not depend on where in the tumour a sample was taken, so it is a candidate predictor that needs no new tumour material.

PD-L1 expression is the one tumour-immune biomarker already used to select patients, so any germline effect has to be read against it. PD-L1 was recorded as tumour proportion score categories (50% or above, 1 to 49% and below 1%; Table 1). Every adjusted model included PD-L1 at 50% or above with treatment type, histology and sex; a second model added ECOG performance status, and PD-L1 and treatment regimen were compared between carriers and non-carriers of the lead allele group (Section 2.1). The question is whether germline HLA or KIR variation is associated with disease control beyond PD-L1 and the clinical covariates. The study was not designed to derive or validate a model for predicting the response to anti-PD-1 or anti-PD-L1 therapy; it asks whether any exposure gives a signal stable enough across endpoints, exposure definitions and an external cohort to justify developing one, and reports the effect sizes that a sample of this size could detect (Table S25).

Here, we examined whether germline *HLA* and *KIR* variation is associated with disease control in a southern Spanish NSCLC cohort treated with immune checkpoint inhibitors, with unfavourable disease status at three months as the primary endpoint. The same patients were classified at each level of aggregation (allele group, supertype, evolutionary divergence, KIR). Genotypes came from whole-exome sequencing, with call resolution audited before any association test. We also benchmarked carrier frequencies against Spanish donor registries and compared the principal class I findings with an external cohort.

## 2. Results

### 2.1. Cohort and Analysis Sets

Ninety-nine patients had both an HLA genotype and an assessable 3-month landmark, 68 with favourable and 31 with unfavourable status (Table 1); genotyped and non-genotyped patients did not differ in any baseline variable (all *p* ≥ 0.16, Table S5).

Each analysis uses the patients with the data it requires, so denominators differ between analyses; every figure and table states its own n, and Figure S1 traces the exclusions and the resulting analysis sets. Three sets are nested: the 99 patients with both an HLA genotype and an assessable 3-month landmark sit within the 102 with an HLA genotype, which sit within the 122-patient cohort. The remaining sets overlap rather than nest: 101 patients had an HLA-A call, and 101 had a KIR genotype, which are not the same 101; 98 had both an HLA genotype and an evaluable time to progression; and 100 had a computable HLA-DQA1 divergence value. The class I analyses include one patient without an HLA-A or HLA-B call, analysed as a non-carrier; excluding that patient changed the A*02 odds ratio from 4.00 to 3.93 and left the B*14 and C*08 odds ratios unchanged at two decimals (0.07 and 0.09).

In the 99 patients of the primary set, median age was 65.9 years and, among those with each variable recorded, 77.9% were men, 63.2% had adenocarcinoma, 22.2% had PD-L1 at 50% or above, 74.2% received chemo-immunotherapy and 48.8% had immune-related adverse events (Table 1; regimens in Table S6). Among the 114 patients with an evaluable time to progression, median TTP was 10.9 months (95% confidence interval [CI] 8.0 to 17.2) with 64 events; median follow-up was 15.4 months by reverse Kaplan–Meier method and the longest observed follow-up was 51.2 months.

ECOG performance status was the only baseline variable that differed between outcome groups (grade 0, 22.1%; grade 1, 71.6%; grade 2 or above, 6.3%; *n* = 95; Fisher *p* = 0.039; Table 1). It was prognostic for time to progression (hazard ratio [HR] 1.81 per grade, 95% CI 1.00 to 3.26, *p* = 0.049; *n* = 94, 54 events), for the composite of progression or death (HR 2.82, 1.69 to 4.72, *p* < 0.001; 78 events) and for 3-month unfavourable status (odds ratio [OR] 3.12 per grade, 95% CI 1.26 to 9.05, *p* = 0.013; *n* = 95).

PD-L1 category did not differ between outcome groups (Fisher *p* = 0.652), and neither did treatment type (*p* = 0.129; Table 1). Among the patients with each variable recorded, PD-L1 at 50% or above was present in 26.9% (7 of 26) of A*02 carriers and 20.3% (13 of 64) of non-carriers (Fisher *p* = 0.578), and chemo-immunotherapy was received by 71.4% (20 of 28) and 75.4% (49 of 65), respectively (*p* = 0.797).

### 2.2. Genotyping Performance at Allele-Group Resolution

Call resolution constrains every association reported below, so the audit comes first. Of 1155 patient-by-locus units, 99.1% resolved at the allele-group level and 94.3% at two fields, with near-complete per-gene coverage across the 12 typed genes (Table S2). The presence-dependent loci yielded lower counts, as expected: *DRB3* (69), *DRB4* (68) and *DRB5* (27). These are call-coverage counts across the audited samples, not the denominators of Table S24, which is restricted to the 102-patient analysis cohort; within that cohort, the numbers of gene-positive patients at *DRB3*, *DRB4* and *DRB5* are 64, 62 and 26.

*KIR* calls behaved differently: 195 of 1025 gene-present units (19.0%) were ambiguous and none resolved at the first field, with ambiguity concentrated in the genes with the closest paralogues (*KIR2DS3* 52.0%, *KIR2DS2* 47.8%, *KIR2DL1* 47.6%), the expected behaviour of a short-read caller in a segmentally duplicated region (Table S2).

### 2.3. Carrier Frequencies and Comparison with Spanish Donor References

Table S24 gives carrier frequencies with 95% intervals for the 70 testable class I and class II allele groups. As a plausibility check, the three groups with the strongest primary-endpoint associations (A*02, B*14 and C*08; Table 2) were compared with two Spanish donor references: a 21,787-donor Catalan bone-marrow registry [28] and 278 bone-marrow donors from Seville, in the same region as this cohort [29]. Both report allele frequencies, so expected carrier frequencies were derived under Hardy–Weinberg equilibrium as 1 − (1 − *p*)^2^, where p is the allele frequency; the Catalan A*02 frequency of 24.08%, for example, gives 42.4% expected carriers. The Catalan values are sums of the alleles listed among the registry’s most frequent and may underestimate the whole group (Table S24), so the expected carrier frequencies from Catalonia are lower bounds. Donors are healthy and self-selected; neither panel is the source population of this cohort, and the comparisons are descriptive and outside the correction structure of the association screen.

Against the Catalan registry, B*14 and C*08 carriage was close to expectation (11.9% against 11.3% and 9.8% against 10.9%; exact binomial *p* = 0.875 and 0.874, respectively). Because the two groups are in near-complete linkage (*phi* = 0.903), this is one observation, not two. The Seville panel reports higher frequencies: C*08 carriage was lower than expected (9.8% against 18.5%, *p* = 0.021) and B*14 carriage was lower but not significantly so (11.9% against 17.8%, *p* = 0.151). A*02 carriage was lower than expected against both references: 30.7% (31 of 101 patients with an HLA-A genotype; 95% CI 22.5 to 40.3) against 42.4% from Catalonia (*p* = 0.020) and 45.6% from Seville (*p* = 0.003). Because the Catalan expectations are lower bounds, the true A*02 deficit against Catalonia is, if anything, larger. Four explanations are possible: chance, departure from Hardy–Weinberg proportions, a real difference between patients with lung cancer and healthy donors, and undercalling by the typing algorithm. These data do not separate them. Only A*02 falls significantly below the Catalan expectations, whereas all three groups sit below the Seville ones; but the two panels also differ substantially from each other, by roughly 60 to 70% for B*14 and C*08, and the Seville estimates rest on 278 donors. Section 2.10 examines undercalling directly.

### 2.4. KIR Gene Content and KIR-HLA Ligand Pairs

No KIR association survived correction at any endpoint (*q* is the *p* value adjusted for the false discovery rate; Section 5.5). The strongest nominal results were KIR2DS3 for lower odds of immune-related adverse events (OR 0.28, 0.09 to 0.78, *n* = 0.170) and KIR2DL2 with a C1 ligand for lower odds of unfavourable 6-month status (OR 0.39, 0.16 to 0.91, *p* = 0.029, *n* = 0.403; 39 carriers among 93 patients). Because C*08 is itself a C1 ligand, the second estimate was checked against C*08 carriage and was attenuated (Tables S7 and S8). Gene frequencies and ligand-pair distributions are consistent with southern European references [30].

### 2.5. HLA-DQA1 Evolutionary Divergence, Bimodality and Lineage Identity

At 12 months, higher HLA evolutionary divergence at HLA-DQA1 was associated with lower odds of unfavourable status among the 75 labelled heterozygotes with an evaluable landmark (OR 0.42 per standard deviation, 0.23 to 0.70, *p* < 0.001, *n* = 0.004). This was the only test to reach a false discovery rate below 0.05 within its own hypothesis family. It is therefore a hypothesis-generating finding: it does not survive correction across all 448 tests (*n* = 0.299, below).

**Figure 1:**
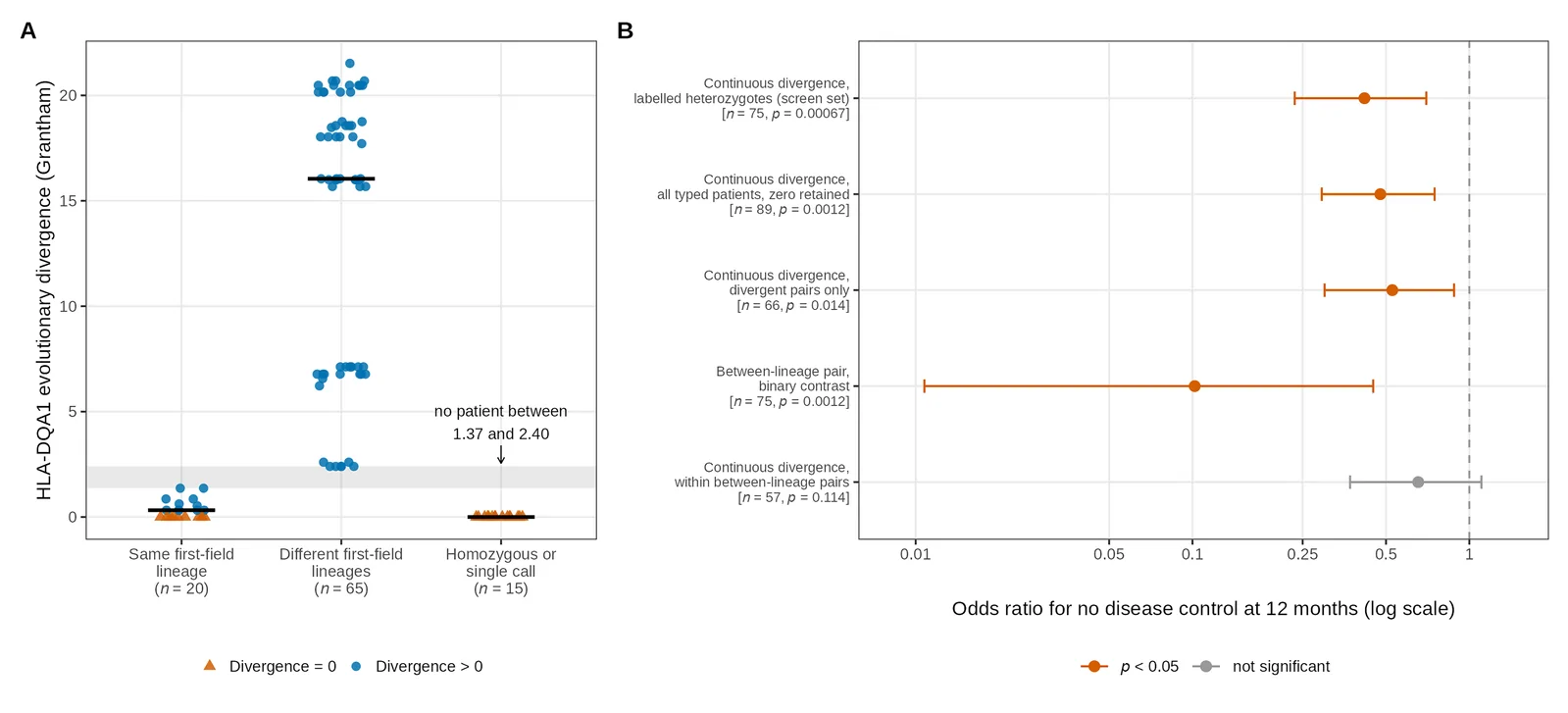
*HLA*-*DQA1* evolutionary divergence is bimodal and tracks first-field lineage identity. (**A**) Recomputed divergence at *HLA*-*DQA1* for 100 of the 102 genotyped patients (two had no computable value), plotted against whether the two alleles belong to the same first-field lineage (*n* = 20) or to different lineages (*n* = 65); the 15 patients labelled homozygous or reported with a single call are shown as a separate stratum. The shaded band marks the interval between 1.37 and 2.40 Grantham units, which contains no patient. (**B**) Odds ratios for no disease control at 12 months from Firth penalised logistic regression under five specifications of the *DQA1* exposure (top to bottom, *n* = 75, 89, 66, 75 and 57): continuous divergence in the labelled heterozygote set, in all typed patients with zero divergence retained and in genuinely divergent pairs only; the binary same-lineage against between-lineage contrast; and continuous divergence within between-lineage pairs only. Continuous estimates are per standard deviation. Bars are 95% profile penalised-likelihood intervals on a logarithmic scale.

Support depends on the analysis set and on the scope of the multiplicity correction (Figure 1, Table S9). Retaining every typed patient with zero divergence gave OR 0.48, *p* = 0.001, and *n* = 0.007 (*n* = 89); restricting to genuinely divergent pairs gave OR 0.53 (0.30 to 0.88), *p* = 0.014, and *n* = 0.084 (*n* = 66); covariate adjustment gave OR 0.50 (0.26 to 0.88), *p* = 0.016, and *n* = 0.098 (*n* = 66). Pooling the four endpoints within the divergence family gave *n* = 0.016, and pooling all 448 tests into a single Benjamini–Hochberg family gave *n* = 0.299 (Figure 1B), so no test in the screen is significant after correction across the whole screen.

Divergence at DQA1 alone does not index the repertoire of the assembled HLA-DQ molecule: polymorphism in both chains shapes the peptide-binding groove [31], and the beta-chain residues have been reported as the more influential of the two [32]. We therefore recomputed divergence at DQB1 as a post hoc completeness analysis. Each model requires computable divergence at every locus it includes, so the analysis sets differ, and the estimates below are not directly comparable to the 0.42 of the screen. DQB1 divergence alone was null at 12 months (OR 0.71 per standard deviation, 0.42 to 1.15, *p* = 0.169, *n* = 77). Among the 65 patients with computable divergence at both chains, DQA1 alone gave OR 0.44 (0.24 to 0.76, *p* = 0.003) and was essentially unchanged when DQB1 was added (OR 0.45, 0.24 to 0.79, *p* = 0.005), while DQB1 itself was flat (OR 0.94, 0.53 to 1.62, *p* = 0.817) (Tables S9c and S9d). The association therefore tracks the alpha chain in these data, which the structural literature on DQ would not predict; with 65 patients, this bounds what can be resolved rather than arguing against a beta-chain contribution.

No other divergence test reached *q* < 0.05: the next smallest was DQA1 at 3 months (OR 0.56, 0.34 to 0.91, *p* = 0.018, *n* = 0.106), with 6 months at OR 0.64 (0.40 to 1.00, *p* = 0.051, *n* = 0.305), and the strongest result at another locus was HLA-A at 3 months (OR 1.51, 0.94 to 2.48, *p* = 0.088, *q* = 0.263; Table S10).

The *DQA1* estimate behaves as a contrast between two groups rather than as a gradient. Recomputed divergence at *DQA1* is bimodal, with an empty interval between 1.37 and 2.40 Grantham units, and the gap coincides exactly with first-field lineage identity: among the 79 of the 85 labelled *DQA1* heterozygotes with both alleles resolved at two fields, the 19 with a same-lineage pair span 0 to 1.37 and the 60 with a between-lineage pair span 2.40 to 21.52 (Figure 1A; restricting to two-field resolution removes one same-lineage and five between-lineage patients from the 20 and 65 shown in the figure). Coded as a binary contrast, the groups separate more sharply: a between-lineage pair gave OR 0.10 for unfavourable status (0.01 to 0.45, *p* = 0.001). Single-call homozygosity cannot be distinguished from dropout, so the contrast is reported in terms of first-field lineage (Figure 1B, Table S9). In the strict two-field set evaluable at 12 months (*n* = 69), 16 of 17 patients with a same-lineage pair had unfavourable status against 28 of 52 with a between-lineage pair (same-lineage OR 9.46, 2.11 to 90.20, *p* = 0.002, the reciprocal of the between-lineage coding used above; Table S9c,f). That restriction excludes dropout among the alleles that did resolve, but not for patients with a single *DQA1* call, who cannot be assigned to either lineage group; the contrast therefore describes first-field lineage and not functional homozygosity. Within between-lineage pairs, the continuous estimate was imprecise rather than absent (OR 0.65, 0.37 to 1.11, *p* = 0.114, *n* = 57), so these data do not separate a pure lineage dichotomy from a dichotomy with a gradient inside it.

### 2.6. The Pre-Specified Screen Across Six Hypothesis Families

The complete screen comprised 448 within-family tests, 112 at each of the four endpoints (Table S10): 36 class I allele groups, 34 class II allele groups, 14 *KIR*-*HLA* ligand pairs, 12 *KIR* gene presence terms, 10 class I supertypes and 6 divergence exposures, each tested at every endpoint. Within families, one test had *q* < 0.05 (*HLA-DQA1* divergence at 12 months); when all 448 tests were corrected as a single family, none did (lowest *q* = 0.299). All 448 tests were carried in the primary analysis; refitting the Benjamini–Hochberg correction on the 433 that met the testability thresholds in their own analysis set leaves the same single within-family result below *q* = 0.05 (Table S10b).

Permutation agrees at the narrower levels but not at the widest: the family-wise error rate for HLA-DQA1 divergence was 0.043 against the 112 exposures tested at 12 months and 0.165 across all 448 exposure-by-endpoint combinations (Table S11). The strongest primary-endpoint result, *HLA*-A*02, had *q* = 0.084 among the 36 class I allele groups.

### 2.7. The Class I Landscape: Two Opposing Allele Groups

**Figure 2:**
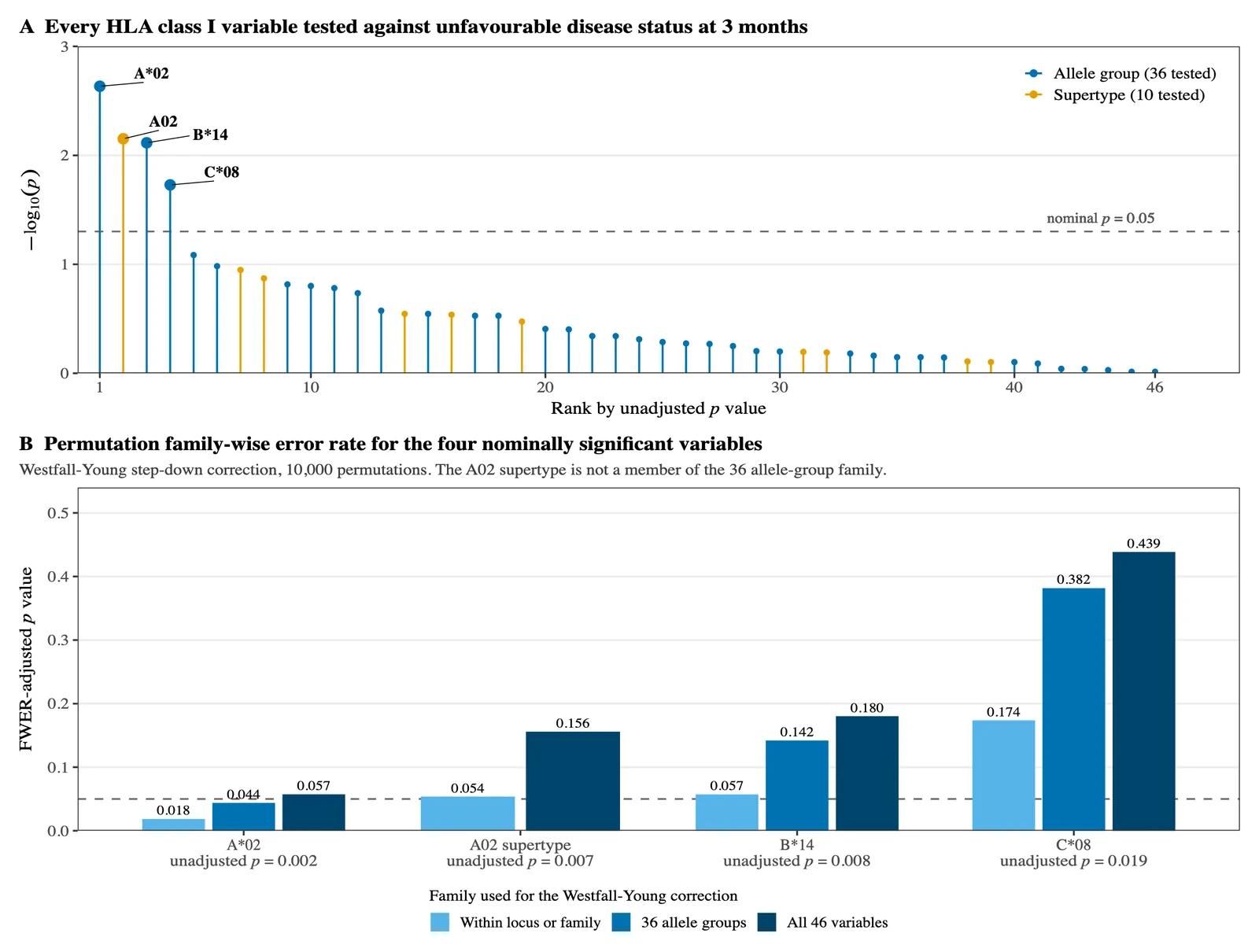
Class I exposure landscape at the primary endpoint. (**A**) Nominal *p* values for all 46 class I exposures (36 allele groups and 10 supertypes) at the 3-month landmark in the 99 patients with an assessable landmark, ordered by significance, with the four nominally significant exposures labelled. (**B**) Permutation family-wise error rates (10,000 permutations) for these four exposures at three nesting levels: within the locus or family, across the 36 allele groups and across all 46 class I exposures.

Among the 46 class I exposures, four reached nominal significance at the 3-month landmark: *HLA*-A*02 (*p* = 0.002), the A02 supertype containing it (*p* = 0.007), *HLA*-B*14 (*p* = 0.008) and *HLA*-C*08 (*p* = 0.019) (Figure 2). B*14 and C*08 were in near-perfect linkage (*phi* = 0.903), reproducing the historical B*14:02–*Cw8* association of Spanish haplotypes [33]. The class I signal therefore reduces to two exposures acting in opposite directions (Tables S14 and S16).

**Figure 3:**
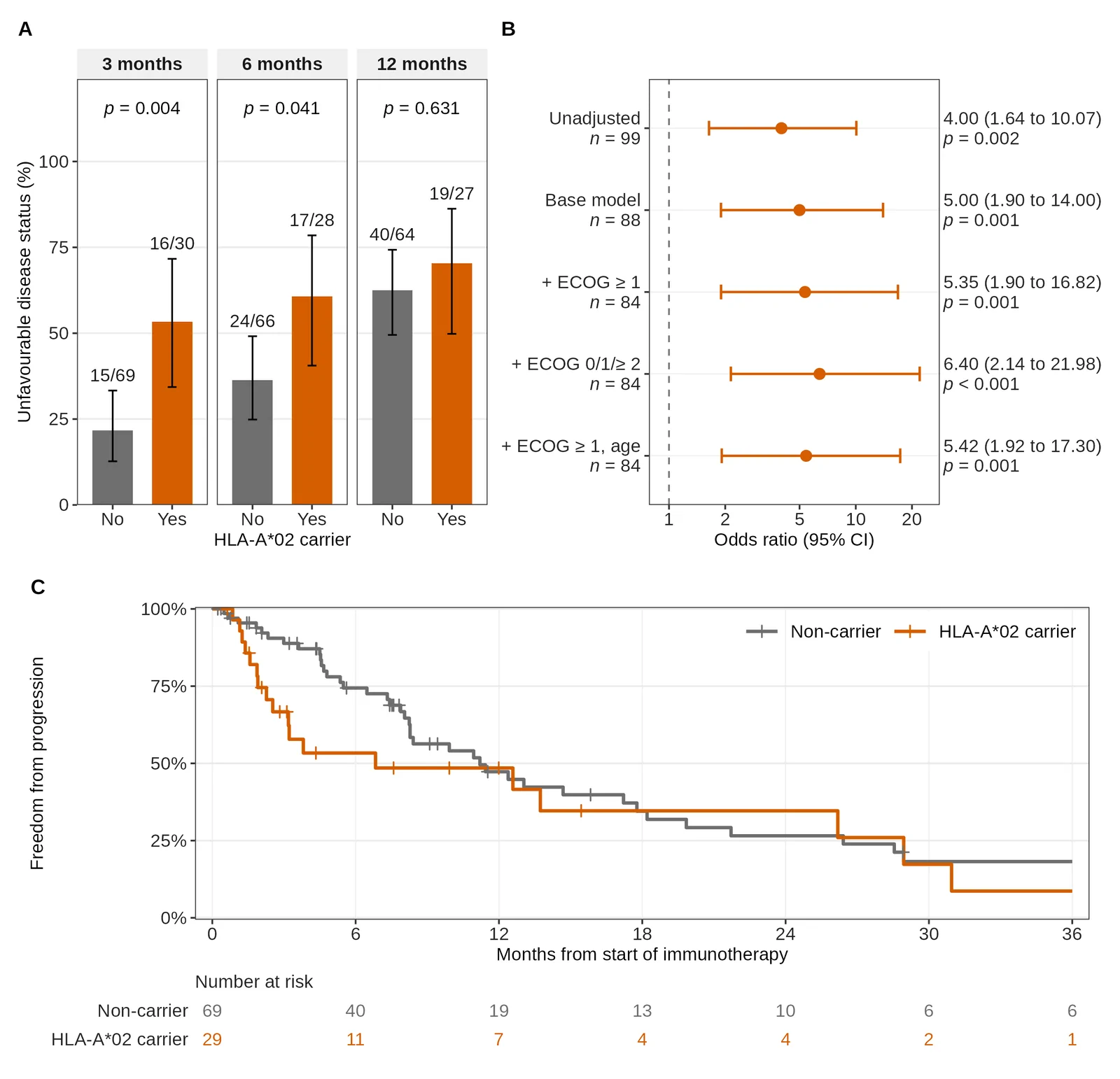
*HLA*-A*02 carriage and early treatment failure. (**A**) Proportion of patients with unfavourable disease status at 3, 6 and 12 months by A*02 carrier status, with sample counts and Fisher’s exact *p* values (0.004, 0.041 and 0.631). (**B**) Odds ratios for unfavourable status at 3 months from Firth penalised logistic regression, with 95% profile penalised-likelihood intervals on a logarithmic scale: the unadjusted model (*n* = 99), the base model with treatment type, PD-L1 (50% or above), histology and sex (*n* = 88), and three models that add ECOG performance status as 1 or above, as three levels (0, 1, 2 or above), or as 1 or above together with age (*n* = 84 each). (**C**) Kaplan–Meier curves of time to progression by A*02 carrier status in 98 patients, with numbers at risk; median is 6.8 against 11.2 months, HR is 1.42 (95% CI 0.81 to 2.49), Cox *p* = 0.217, and log-rank *p* = 0.215.

*HLA*-A*02 carriage (30.3%, 30/99) was associated with unfavourable disease status at 3 months (unadjusted OR 4.00, 95% CI 1.64 to 10.07, Firth *p* = 0.002; Fisher exact *p* = 0.004, Table 1). The association was not attenuated by adjustment for clinical covariates (OR 5.00, 1.90 to 14.00, *p* = 0.001, *n* = 88) and held after adding a binary ECOG term (OR 5.35, 1.90 to 16.82, *p* = 0.001, *n* = 84; Table 2, Figure 3B).

The association attenuated at 6 months (OR 2.64, 1.09 to 6.61, *p* = 0.031) and was no longer distinguishable from the null at 12 months (OR 1.39, 0.55 to 3.72, *p* = 0.495). This early landmark risk did not translate into a time-to-event difference: median TTP was 6.8 months in carriers against 11.2 in non-carriers (HR 1.42, 0.81 to 2.49, *p* = 0.217; *n* = 98) and the composite gave 3.2 against 8.3 months (HR 1.29, 0.80 to 2.06, *p* = 0.297), with estimates unchanged after adjustment and across the three progression event definitions (Table S13).

HLA-B*14 was present in 12.1% of patients (12/99). No B*14 carrier had unfavourable status at 3 months (0/12 against 31/87; Firth OR 0.07, 0.0006 to 0.58, *p* = 0.008; adjusted OR 0.08, 0.0006 to 0.67, *n* = 84). The lower limits are close to zero because no carrier had unfavourable status; they are profile penalised-likelihood limits and are given to four decimals so that they do not round to zero. The protective association was maintained at 6 and 12 months with attenuation (OR 0.14, 0.01 to 0.62, *p* = 0.007, *q* = 0.253, *n* = 94 at 6 months; OR 0.18, 0.04 to 0.63, *p* = 0.007, *q* = 0.078, *n* = 91 at 12 months; Table S10). No B*14 estimate reached *q* < 0.05 at any endpoint.

**Figure 4:**
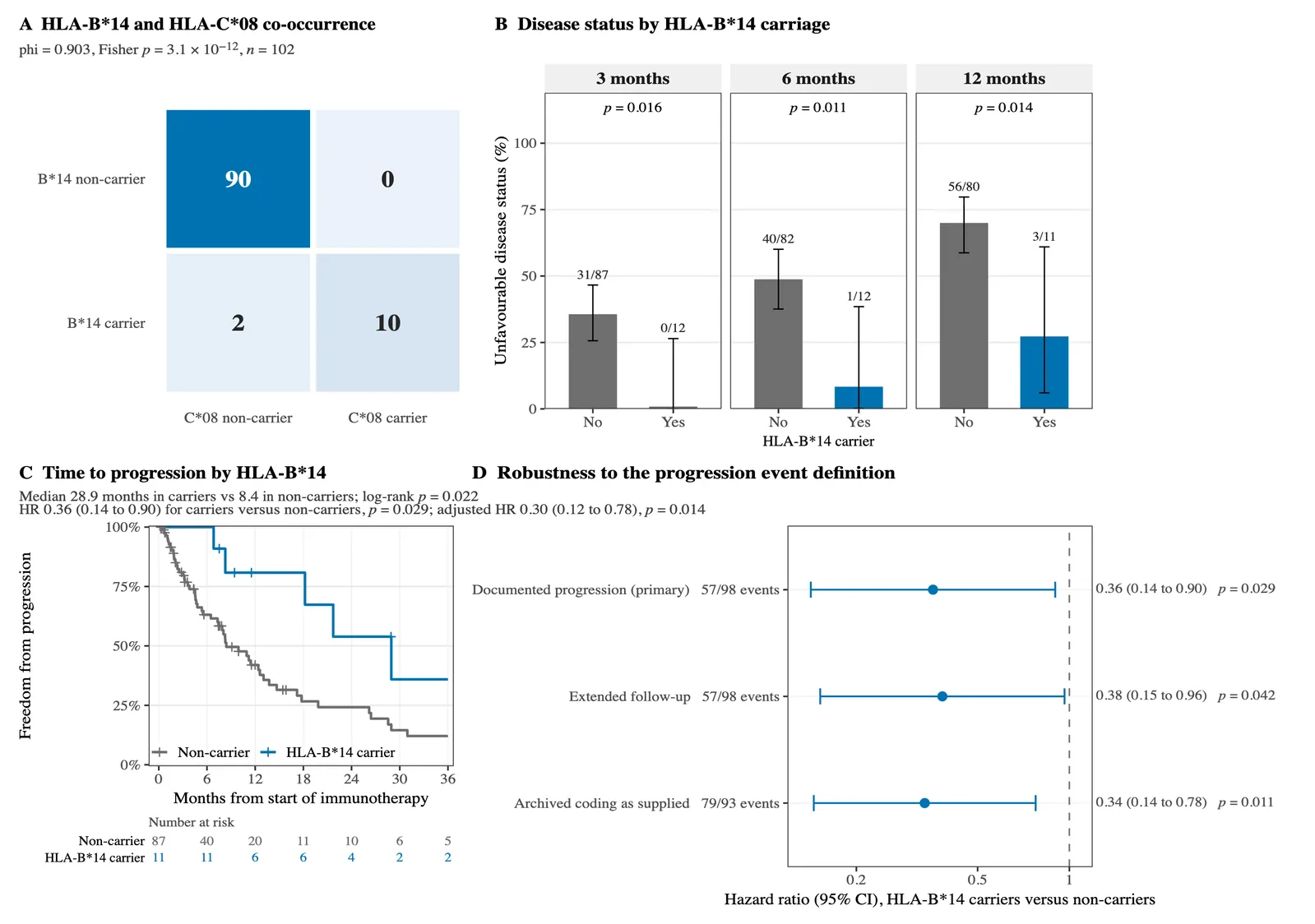
*HLA*-B*14 carriage and treatment response. (**A**) Cross-tabulation of B*14 and C*08 carriage in the 102 genotyped patients (*phi* = 0.903, Fisher *p* < 0.001). (**B**) Proportion of patients with unfavourable disease status at 3, 6 and 12 months by B*14 carrier status (0.0% in carriers at 3 months), with sample counts and Fisher’s exact *p* values. (**C**) Kaplan–Meier time to progression curves by B*14 carrier status in 98 patients, with numbers at risk. (**D**) Hazard ratios for B*14 carriage under the three progression event definitions: documented progression, extended follow-up and archived coding (Table S13).

B*14 carriage was also associated with longer time to progression (median 28.9 against 8.4 months; HR 0.36, 0.14 to 0.90, *p* = 0.029) and with the composite of progression or death (HR 0.37, 0.17 to 0.81, *p* = 0.013; median 21.7 against 6.5 months; Table 2, Figure 4). Its linked partner C*08 gave consistent but attenuated estimates (Table 2).

The two markers were statistically unlinked (*phi* = −0.109, *p* = 0.337; only 2 patients carried both) and retained opposing associations under mutual adjustment (A*02 OR 3.77, 1.50 to 9.80; B*14 OR 0.08, 0.0006 to 0.67), but with two double carriers and complete outcome separation, these estimates are arithmetic consequences of the penalisation rather than evidence of biological independence.

In a post hoc ordered three-level classification (B*14 carrier, neither, A*02 carrier), the two patients carrying both markers were excluded. Unfavourable status at 3 months rose monotonically: 0.0%, 25.4% and 57.1% (trend OR 4.53, 2.02 to 11.07, *p* < 0.001), and median TTP declined from 28.9 to 9.9 to 3.8 months (HR 1.67, 1.09 to 2.56); the gradient is descriptive, and robustness analyses left both estimates intact (Tables S16 and S23, Figure S2).

Genetic ancestry and smoking history were examined in sensitivity analyses (Table S26). Ancestry scores were available for 98 of the 99 patients. In a principal component analysis of the scores (components 1 and 2, 80.3% and 13.8% of the variance), 87 patients formed a main cluster, and 11 lay outside it (Figure S6). Six of the 11 carried A*02, against 24 of 87 in the main cluster (Fisher *p* = 0.087), and neither component differed between carriers and non-carriers or between outcome groups (Wilcoxon *p* of 0.37 or more). The A*02 odds ratio, 3.93 (1.61 to 9.89) in the 98 patients, was 3.57 (1.44 to 9.02, *p* = 0.006) after adjustment for position outside the main cluster and 3.78 (1.54 to 9.58, *p* = 0.004) after adjustment for the two components, and was lower, 2.91 (1.09 to 7.85, *p* = 0.033; *n* = 87), when the analysis was restricted to the main cluster. B*14 was 0.06 and 0.08 after the two adjustments (*p* = 0.004 and 0.010) and 0.10 after restriction (*p* = 0.027). C*08, which is in near-complete linkage with B*14 (*phi* = 0.903), was 0.08, 0.10 and 0.11 (*p* = 0.017, 0.024 and 0.041), but *p* was 0.091 after restriction with clinical covariates (8 carriers) and 0.051 after restriction with the two components.

Smoking status was recorded for 68 patients (38 current, 27 former, 3 never smokers) and pack-years for 63. Neither current smoking (Fisher *p* = 1.000) nor pack-years (Wilcoxon *p* = 0.185) was associated with unfavourable status. Current smoking was not associated with A*02 or B*14 carriage (*p* = 0.623 and 1.000), but A*02 carriers had fewer pack-years than non-carriers (*p* = 0.037, or 0.055 without the two lower-bound values). In these 68 patients, the A*02 odds ratio was 10.33 (3.32 to 36.71) and changed little after adjustment for current smoking (10.12) or, in 63 patients, for pack-years (8.58, 2.63 to 31.80). In a combined model with clinical covariates, current smoking and ancestry (*n* = 64, 19 events), the A*02 odds ratio was 8.94 (2.70 to 35.59) with the indicator of position outside the main cluster and 7.98 with the two components; B*14 was 0.07 and 0.15 (*p* = 0.015 and 0.102) and C*08 was 0.10 and 0.18 (*p* = 0.055 and 0.160). Both models have fewer than three events per predictor. Status was missing for 31 patients, in whom A*02 was less frequent (5 carriers, against 25 of 68; Fisher *p* = 0.058) and its odds ratio was 0.52 (0.05 to 3.38), different from that in the patients with status (post hoc interaction *p* = 0.007).

### 2.8. Supertypes and Allele Groups Are Different Exposures

Supertypes and allele groups were pre-specified as separate hypothesis families, and at two loci the two definitions gave discordant answers.

At *HLA*-*B*, the B27 supertype was null across all 29 carriers at 3 months (OR 0.80, 0.30 to 1.99) and for TTP (HR 0.75, 0.41 to 1.36), despite containing B*14. For its 12 B*14 carriers alone, against all other patients, the association was pronounced (OR 0.07, 0.0006 to 0.58), whereas the 17 remaining B27 carriers, against non-carriers of B27, showed the opposite direction (OR 1.81, 0.62 to 5.21; Table S14). The three-strata decomposition is post hoc (Figure S3).

At *HLA*-*A*, the A02 supertype (35 carriers) gave a 3-month OR of 3.29 (1.38 to 8.05) against 4.00 for A*02 alone, and the five supertype carriers lacking A*02 gave an OR of 0.70 (0.07 to 4.03). The permutation family-wise *p* value was 0.018 for A*02 within *HLA*-*A* but 0.054 for the A02 supertype within the supertype family (Figure 2B).

### 2.9. Class II: Resolution Determines Visibility

No class II association survived correction (minimum *n* = 0.207). Three linked markers were nominally associated with worse 6-month status (DRB1*11, OR 3.27, 1.23 to 9.30; DQB1*03:01, OR 3.26, 1.34 to 8.32; and DQA1*05, OR 2.69, 1.18 to 6.28), and on time to progression DQB1*03:01 gave the strongest class II estimate (HR 2.21, 1.26 to 3.85, *p* = 0.005; Table S15, Figure S4).

On time to progression, DQB1*03:01 carriers had a median of 5.5 months against 14.7 months in non-carriers. The hazard ratio was 1.91 (1.03 to 3.54, *p* = 0.041) after clinical adjustment and, with the three linked markers mutually adjusted, DQB1*03:01 retained an elevated hazard ratio of 2.15 (0.92 to 5.02, *p* = 0.078) while DRB1*11 collapsed to the null (HR 1.00, 0.38 to 2.62). This is the estimate most sensitive to the event definition: on the composite of progression or death, the unadjusted hazard ratio attenuates to 1.63 (1.01 to 2.64, *p* = 0.045), clinical adjustment leaves 1.40 (0.82 to 2.39, *p* = 0.222) and the mutually adjusted estimate falls to 1.20 (0.55 to 2.62, *p* = 0.640), with DRB1*11 at 1.56 (0.65 to 3.76, *p* = 0.322). DQB1*03:01 therefore withstands clinical adjustment under documented progression but not under the composite, and under neither definition is it separated from its linked markers once they are mutually adjusted (Table S15).

The class II signal was confined to the two-field allele, the resolution at which DQB1*03:01 was pre-specified for analysis (Section 5.3): the first-field group DQB1*03 (46 carriers, including the 28 with DQB1*03:01) was null at 6 months (OR 1.17, 0.52 to 2.64, *p* = 0.700) and on both time-to-event definitions (time to progression HR 0.97, 0.58 to 1.64, *p* = 0.912; composite HR 0.84, 0.54 to 1.29, *p* = 0.421). The decomposition below uses patients carrying no DQB1*03 allele as the reference, which is not the reference used earlier in this section; the estimates for DQB1*03:01 are therefore not comparable with the OR 3.26 quoted above. Against that reference, the 18 carriers of DQB1*03 without DQB1*03:01 ran in the protective direction (6-month unfavourable status OR 0.31, 0.07 to 1.05, *p* = 0.060, *n* = 66; time to progression HR 0.43, 0.19 to 0.96, *p* = 0.039, *n* = 68; composite HR 0.46, 0.24 to 0.88, *p* = 0.019, *n* = 68), while DQB1*03:01 ran the other way (OR 2.45, 0.96 to 6.48, *p* = 0.060, *n* = 76; time to progression HR 1.70, 0.95 to 3.02, *p* = 0.072, *n* = 80; composite HR 1.31, 0.80 to 2.16, *p* = 0.281, *n* = 80). Only the two time-to-event estimates in the 18-carrier stratum reach nominal significance; all are post hoc, unadjusted and bounded by 18 carriers (Table S15b). Opposite effects within the first-field group would produce the null seen for the pooled DQB1*03 group. This is the resolution-level counterpart of the supertype dilution in Section 2.8: in both, pooling averages effects that point in opposite directions, so the level at which an exposure is defined determines whether an association is visible, not whether it exists.

### 2.10. Comparison of the Class I Signals with an Independent Public Cohort

All internal estimates in this section are fitted on the 95 patients with a class I genotype, an evaluable 3-month status and an evaluable time-to-progression record under the external protocol (Table S20b), and all external estimates on the 297 of 309 SU2C-MARK patients evaluable for progression-free survival (289 for the 90-day landmark, set against the 99-patient primary analysis). Estimates on the larger internal sets used elsewhere in this paper are given in Table S20. Table S20b gives the estimates on these 95 patients under both time-to-event definitions.

We examined our two strongest signals in SU2C-MARK, an independent public cohort of checkpoint-treated advanced NSCLC, using its published POLYSOLVER germline class I calls; the B*14 association was not supported, the A*02 time-to-event comparison was inconclusive, and the A*02 association at 3 months was not reproduced on a 90-day landmark. B*14 and the B27 supertype were present at similar frequencies in the two cohorts, so prevalence does not limit the comparison; A*02 carriage was more frequent externally (42.4% against 30.3% in the primary set), a gap of the same size as the deficit against the Spanish donor references (Tables S20 and S24).

The lower internal A*02 frequency does not account for the discrepancy (Tables S18 and S19), and two observations argue against a calling artefact: B*14 and C*08, which are in near-complete linkage and therefore count once, match the Catalan reference while only A*02 is deficient; and carriage did not track call quality, with the extreme reclassification, which raises carriage to 39.4% against the registry value of 42.4%, still leaving an odds ratio of 2.49 (1.06 to 5.97). Residual undercalling would in any case be non-differential and bias toward the null (Table S18c).

Among the 297 externally evaluable patients, B*14 showed no protective association. Against all other patients, the external progression-free survival HR was 1.14 (0.72 to 1.78) compared with an internal time-to-progression HR of 0.36 (0.14 to 0.89), formally rejecting a homogeneous effect (Cochran *q* *p* = 0.027, I^2^ 79.7%, the proportion of the variability between the two estimates that reflects true differences rather than chance; Section 5.6). Restricting both cohorts to B27 carriers, the external HR was 1.17 (0.70 to 1.97) against an internal 0.26 (0.09 to 0.77) for time to progression (Q *p* = 0.015, I^2^ 83.3%) and against an internal 0.31 (0.12 to 0.77, *p* = 0.012) for the composite of progression or death (Q *p* = 0.013, I^2^ 83.8%; Table S20b). The external estimate is identical in the last two comparisons, which differ only in the internal endpoint definition.

**Figure 5:**
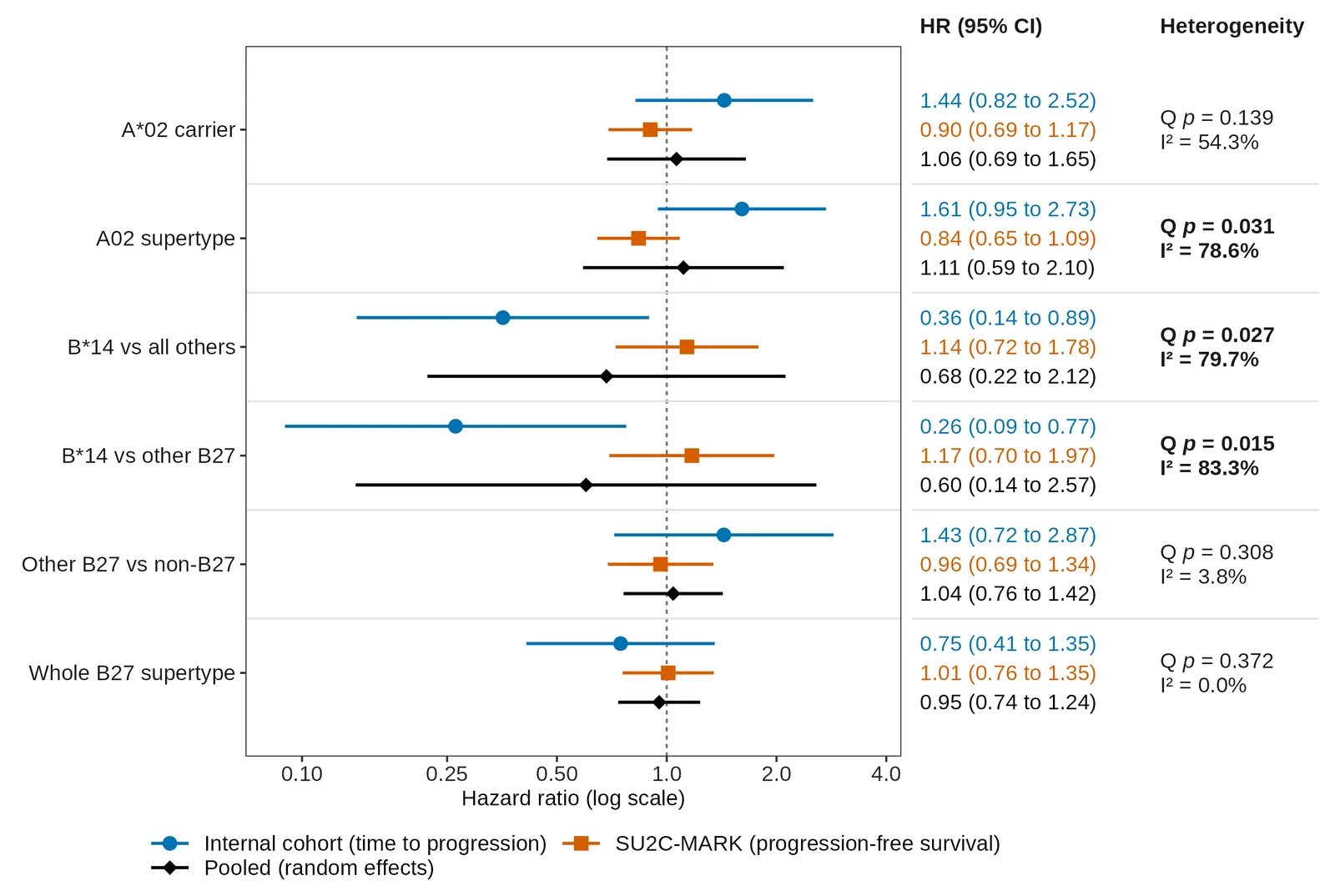
Compatibility of the two class I signals with an independent public cohort. Forest plot of unadjusted Cox hazard ratios (HR) with 95% confidence intervals for the pre-specified contrasts, for time to progression in this cohort (95 patients, 57 events) and for progression-free survival in SU2C-MARK (297 patients, 232 events), with DerSimonian–Laird pooled estimates. The right-hand columns give the *p* value of Cochran Q, which tests whether the effect is the same in both cohorts, and I^2^, the proportion of the variability between estimates that reflects true differences between cohorts; *q* *p* values below 0.05 are in bold. The x-axis is logarithmic, and the dashed line marks the null. Internal estimates are fitted on the 95 patients with a class I genotype, an evaluable 3-month status and an evaluable time-to-progression record under the external protocol (Table S20b).

For A*02, the external HR was 0.90 (0.69 to 1.17) against 1.44 internally, and homogeneity was not rejected under either endpoint definition (time to progression *q* *p* = 0.139, I^2^ 54.3%; composite of progression or death *q* *p* = 0.195, I^2^ 40.6%), so the A*02 time-to-event comparison is inconclusive rather than discordant. On the pre-specified 90-day landmark, the external analogue of the primary endpoint (289 assessable patients, 101 unfavourable), A*02 carriage was not associated with early failure (OR 0.77, 0.47 to 1.25, against 4.00 internally; Cochran *q* *p* = 0.002, I^2^ 89.9%) and B*14 carriage was not protective (OR 1.25, 0.55 to 2.71, against 0.07 internally; Table S20c). The 3-month A*02 signal was therefore not reproduced on this endpoint, although the landmark is derived from follow-up and is not the clinician-assigned status. The A02 supertype, which contains A*02, did show heterogeneity (HR 1.61, 0.95 to 2.73 internally against 0.84, 0.65 to 1.09 externally; time to progression *q* *p* = 0.031, I^2^ 78.6%; composite of progression or death *q* *p* = 0.076, I^2^ 68.2%): a supertype can be less consistent across cohorts than the allele group it contains, because the frequencies of its remaining members need not match between populations. For time to progression, the nulls were consistent across cohorts for the whole B27 supertype (pooled HR 0.95, 0.74 to 1.24, I^2^ 0.0%, *q* *p* = 0.372) and for B27 carriers other than B*14 against non-carriers of B27 (Q *p* = 0.308, I^2^ 3.8%; Figure 5, Table S20b).

The two cohorts diverge in treatment context: in SU2C-MARK, almost no patient received chemo-immunotherapy (1 of 309 received PD-(L)1 blockade with an agent other than CTLA-4 blockade, against 74.2%, 69 of 93, internally), 60.2% received treatment beyond the first line, and 20.7% received combinations including cytotoxic T-lymphocyte antigen 4 (CTLA-4) blockade. These data cannot resolve whether the B*14 discrepancy reflects these clinical differences or the absence of a true biological effect: the B*14 contrasts reject a single effect common to both settings, but they do not exclude effect modification by treatment line or regimen. Incorporating a previously reported first-line B27 supertype estimate [18] into a three-cohort meta-analysis likewise rejected a single underlying effect (Q *p* = 0.019, I^2^ 75.7% from restricted maximum likelihood; Table S20), although heterogeneity statistics computed from three small studies are themselves imprecise; the external comparison is described in Table S20, and its frozen protocol is deposited with the analysis code.

### 2.11. Reconstructed Overall Survival

Reconstructed overall survival was a secondary exploratory endpoint (Supplementary Methods, Section S3; Table S21): among 98 patients, there were 76 deaths and median survival was 12.2 months. The median time to progression of 10.9 months in Section 2.1 was estimated in 114 patients, and counts documented progression only, so the 25 of 76 deaths without documented progression are censored; in these 98 patients, the medians were 11.2 months for time to progression, 7.9 months for the composite of progression or death and 12.2 months for overall survival (Table S13i). The 448-test screen was not repeated for survival. Four exposures were instead carried forward after the primary analyses (DQA1 lineage from the divergence family, and A*02, B*14 and C*08 as the strongest carrier signals at the primary endpoint), with Holm adjustment applied within them; because this family was selected once the main results were known, the adjustment is descriptive rather than a pre-specified correction. Same-lineage status at DQA1 was nominally associated with higher mortality (HR 1.92, 1.08 to 3.43; raw *p* = 0.027, adjusted *p* = 0.109; *n* = 82 with 61 deaths, the patients assignable to a lineage group) and B*14 carriage with longer survival (HR 0.42, 0.19 to 0.93; raw *p* = 0.033, adjusted *p* = 0.109; *n* = 98 with 76 deaths); A*02 and C*08 were not (Figure S5). These are exploratory analyses, not confirmatory.

## 3. Discussion

We screened germline HLA and KIR variation against disease control in 122 checkpoint-treated patients with advanced NSCLC, testing 448 pre-specified exposure-by-endpoint combinations across six hypothesis families. No association at the primary endpoint met the declared within-family threshold, and no test survived correction across all 448 tests (lowest *n* = 0.299). One test, *HLA-DQA1* divergence at 12 months, did reach the within-family threshold. It is a hypothesis-generating secondary finding, and its support depended on how the exposure was defined and on how widely multiplicity was corrected; the exposure proved bimodal, so it contrasts two lineage groups rather than grading a repertoire. Of the two strongest class I estimates, the B*14 association was not supported in an independent cohort, and A*02 was inconclusive for time to event and not reproduced at a 90-day landmark. What this screen establishes is therefore methodological rather than clinical: the level at which an HLA exposure is defined determines whether a signal is visible at all.

The most transferable observation concerns exposure definition. Supertypes and allele groups are different molecular objects: alleles sharing a predicted binding motif still differ in the peptide repertoires they present [34]. In this cohort, the B27 supertype was null while its B*14 carriers gave a large protective estimate, and DQB1*03 was null while DQB1*03:01 was not. A study reporting only supertypes will under-detect allele-specific effects; a study reporting only allele groups pays a heavier multiplicity price. Pre-specifying both families and correcting across their union is what makes cohorts comparable.

HLA-A*02 was the strongest class I result at the primary endpoint, with permutation family-wise error rates of 0.018 within HLA-A, 0.044 across the 36 class I allele groups and 0.057 across all 46 class I exposures; all three are correct answers to different questions, and together they make it the strongest class I signal rather than a validated one. Its carrier frequency was also lower than both Spanish donor references (30.7%, 31 of 101 patients with an HLA-A call, against 42.4% and 45.6%), which bears on how the estimate should be read. Undercalling is the obvious explanation, and the pattern does not fit it: B*14 and C*08 match the Catalan reference closely while only A*02 is deficient, carriage did not track call quality, and the most extreme reclassification, which raises carriage to 39.4% against the registry value of 42.4%, still leaves an odds ratio of 2.49 (1.06 to 5.97). Chance and a genuine difference between Andalusian patients and Catalan donors remain open; the frequency reported here should not be taken as a population estimate.

The external comparison bounds how far either signal can be taken. In 297 independently genotyped patients, the B*14 association was not supported, and a homogeneous effect was formally rejected, while A*02 was inconclusive for time to event and not associated with early failure at the 90-day landmark (Section 2.10). Two readings remain. The internal effects could be specific to chemo-immunotherapy, which almost no SU2C-MARK patient received; or they could reflect selecting the most extreme of 46 exposures in 99 patients. The second is more parsimonious, and A*02 and B*14 should be read as cohort-specific descriptive findings.

If either class I association is real, two mechanisms documented for these molecules in other settings would be candidates, and neither was measured here. B*14:02 restricts a CD8+ T cell response that is unusually effective in HIV control, and that effectiveness tracks the functional avidity of the response rather than the protein specificity (Env versus Gag) of the response [35]; in tumours, the avidity of CD8+ clones has likewise been linked to their efficacy [36], so the observation is not obviously confined to viral control. Separately, C*08:02 is a C1 ligand, so B*14~C*08 carriers are educated through the C1 axis [21,37], which is also the axis of the nominal KIR2DL2 with C1 ligand association reported in Section 2.4. Testing either mechanism would require tetramer-sorted receptor repertoires or NK functional assays, which this cohort does not provide.

The magnitude of the A*02 estimate is that of a selected maximum. Across 2000 permutations of the outcome label, the largest odds ratio among the 17 class I exposures with carrier frequency comparable to A*02 had a median of 2.77 and a 95th percentile of 6.09 under a complete null; across all 46 exposures, where rare groups dominate the maximum, the median rose to 7.36 (Table S22). The observed 4.00 is 1.4 times the frequency-matched null median and falls below its 95th percentile, so it should be read as an estimate that selection has probably inflated. It is also discordant across endpoints: OR 4.00 at 3 months against hazard ratios of 1.29 to 1.42 with intervals crossing the null, and no association with overall survival (HR 1.36, 0.84 to 2.21; Table S21), an interval too wide to demonstrate an exclusively early effect. A genuine early effect that does not persist would produce this profile, and so would selection; these data do not separate them, and A*02 is reported here as a description of early failure in this cohort rather than as a measure of progression risk.

In restricted mean survival time at 24 months, treatment interruption (−8.9 months) and chemo-immunotherapy (+5.6 months) outranked every *HLA* or *KIR* estimate (Table S17). Treatment interruption partly reflects deterioration (reverse causation), and clinical variables and fixed germline exposures are not directly comparable, so this ordering gives context rather than a causal ranking 

The first of several limitations is statistical power. The primary analysis rests on 99 patients, 31 with unfavourable status at 3 months. For an exposure carried by 30% of patients, such as A*02, the smallest odds ratio detectable with 80% power is 3.76 (or 0.20 in the protective direction) at a two-sided significance level of 0.05, and 9.49 with a Bonferroni correction for 448 tests. For an exposure carried by 12%, such as B*14, it is 6.34 at the nominal level, and a protective association did not reach 80% power at any odds ratio down to 1/400 (Table S25). This is because about one sample in four of this size would contain fewer than 10 carriers, too few for even zero unfavourable outcomes among them to reach *p* < 0.05; B*14, with 12 carriers, fell on the detectable side of that threshold. The observed A*02 odds ratio of 4.00 therefore lies close to the detection limit and has probably been inflated by selection (Table S22). The B*14 estimate rests on 12 carriers with no unfavourable outcome, and the *DQA1* contrast on 19 patients with a same-lineage pair. Allele groups with fewer than 5 carriers were not tested, so rarer alleles and KIR variants carried by few patients were out of reach. The adjusted models are thin on the same count, about 4.5 to 5.6 events per variable, so they are sensitivity analyses rather than better-specified replacements, and the two-marker gradient was derived from the data it describes (Table S23). Only large effects could be detected, and the absence of an association for other exposures is not evidence that no effect exists.

Multiplicity is the second limitation. No test reached *q* < 0.05 when all 448 were corrected as one family (lowest *n* = 0.299). The within-family *q* of 0.004 for *HLA-DQA1* divergence answers a narrower question: whether that exposure stands out among the six divergence exposures at one endpoint. Its permutation family-wise error rate rises from 0.043 across the 112 exposures tested at 12 months to 0.165 across all 448 combinations (Table S11). The nominal associations for A*02, B*14 and C*08 should be read the same way, as descriptions of this cohort that selection among many exposures may have inflated.

The endpoint is the third limitation. Favourable status at 3, 6 and 12 months groups response with stable disease, and unfavourable status groups progression with death, so it does not record when progression occurred (Section 5.2). Status came from routine clinical assessment without central radiological review. The A*02 signal at 3 months was not reproduced in time to progression (HR 1.42, 0.81 to 2.49) or in progression or death (HR 1.29, 0.80 to 2.06).

Tumour mutational burden (TMB), tumour HLA loss of heterozygosity (LOH) and TNM substage were not available, because only germline exome data were generated. TMB and LOH, which are frequent in NSCLC [25], could be mediators or effect modifiers of a germline HLA effect rather than confounders, so adjusting for them would change the question; their absence means that no association reported here can be attributed to a mechanism. Ancestry and smoking history were examined in sensitivity analyses (Section 2.7, Table S26). Ancestry is the most plausible route to confounding, because HLA allele frequencies differ between populations. The A*02 association stayed nominally significant after adjustment (odds ratios 3.6 to 4.7) and restriction to the main cluster (2.9, *p* = 0.033), and B*14 stayed below 0.12 and C*08 below 0.15, although *p* for C*08 was 0.091 and 0.051 in two restricted analyses. Finer structure within the main cluster cannot be excluded, because ancestry was inferred from genotypes imputed from exome reads and summarised by two components. HLA class II heterozygosity is associated with lower lung cancer risk in smokers [6] and smoking history with higher TMB [38], and adjustment for current smoking or pack-years left the class I point estimates close to those without it, although in the smaller subsets C*08 and, with pack-years, B*14 no longer reached *p* < 0.05. A combined model with ancestry and current smoking (64 patients, 19 events, fewer than 3 events per predictor) kept the A*02 odds ratio above 7, but C*08 no longer reached *p* < 0.05 and B*14 did so only with the cluster indicator (Table S26). Smoking data were missing for 31 of the 99 patients, mainly those who started treatment in 2023, and in these patients A*02 was not associated with outcome (5 carriers; post hoc). The subset with data is therefore not representative, and these analyses cannot show that smoking is irrelevant in patients without data.

Call resolution is a molecular constraint on what can be analysed, not a reporting detail (Section 5.3 and Section 2.2). HLA resolved well at allele-group and two-field level; KIR did not, with ambiguity concentrated in the genes with the closest paralogues and no gain from collapsing to the first field. Both regions combine extreme polymorphism with paralogy, which is why alignment to a single reference followed by variant calling neither reconstructs HLA allele groups nor establishes KIR gene content. Exome reuse therefore costs no additional sequencing [39] but is interpretable only alongside an audit of what the calls will bear, which for KIR means gene presence and little more. The external comparison also sets exposures called by two different methods against each other, T1K on exome data internally and POLYSOLVER externally, so the comparison between cohorts mixes two typing approaches whose discordance was not quantified.

Two measurement constraints bound the exposures themselves. The class I exposure is a haplotype rather than an allele (A*33:01~C*08:02~B*14:02~DRB1*01:02~DQB1*05:01 is the fifth most frequent five-locus haplotype in the Catalan registry [28]), so any causal allele is inherited with the others and cannot be isolated here. At DQA1, nine patients had a single call, and one had none, so neither group could be assigned to a lineage, and a further seven carried two identical calls and falls outside the heterozygote contrast; these 17 exclusions are what leave the 85 heterozygotes analysed in Section 2.5 (Table S9i).

Treatment was heterogeneous (74.2% chemo-immunotherapy, 25.8% ICI monotherapy), although PD-L1 and regimen were balanced across A*02 carriage and entered the adjusted models. Every patient was recruited in a single region of southern Spain, and 87 of the 98 patients with ancestry scores belonged to a single main cluster (Table S26, Figure S6), so the carrier frequencies that determine power here will not transfer directly to other populations.

## 4. Conclusions

In this exploratory NSCLC cohort, no primary-endpoint association met the declared correction threshold, and no test in the screen survived correction across all 448 tests. *HLA*-*DQA1* lineage-associated divergence generated a secondary hypothesis, but its support depended on how the exposure was defined and on how widely multiplicity was corrected; in an independent cohort, the B*14 association was not supported while A*02 was inconclusive for time to event and not reproduced at a 90-day landmark. The study had power only for large effects (Table S25).

The constraint behind all of it is molecular. Exome capture supports *HLA* analysis at allele-group resolution but not *KIR* allele assignment, and aggregation is not a neutral summary at either level: the B27 supertype concealed an opposing pair of allele-group effects, and the first-field group DQB1*03 concealed the signal carried by DQB1*03:01.

A report that fixes one level of aggregation without showing the others cannot tell a reader whether a null is a null. Pre-specifying both exposure families, correcting across their union and publishing the genotype landscape beside the association are what make a cohort of this size worth pooling. Independent validation is required before any claim of clinical utility.

## 5. Materials and Methods

### 5.1. Study Design, Setting and Participants

This germline *HLA* and *KIR* study is a secondary, retrospective analysis of a prospective, multicentre institutional cohort of patients treated with immune checkpoint inhibitors (ICI) reported according to STROBE [40]. Adults with histologically confirmed stage IIIB/IV NSCLC who started anti-PD-1 or anti-PD-L1 therapy between January 2018 and June 2024 at two Málaga hospitals (Spain) were eligible; prior ICI exposure and another malignancy within five years were exclusions. All patients gave written informed consent.

Of 131 patients in the clinical database, we excluded 6 with no retrievable clinical record, 2 with small cell histology, and 1 screening failure, leaving 122 (Figure S1). Age and Eastern Cooperative Oncology Group (ECOG) performance status were recovered from archived records (Table S1). Germline DNA yielded *HLA* genotypes for 102 patients and *KIR* genotypes for 101; 114 had an evaluable time to progression (TTP). The primary analysis set, patients with both an *HLA* genotype and a recorded 3-month disease status, comprised 99 patients.

### 5.2. Endpoints

Disease status at 3, 6 and 12 months after the first ICI dose was classified as favourable (complete or partial response or stable disease) or unfavourable (progressive disease or death) using RECIST 1.1 [41] as recorded in routine clinical assessment, without central radiological review; death before a timepoint counts as unfavourable. Status was known for 118 of the 122 patients at 3 months, 111 at 6 months and 106 at 12 months. Unfavourable status at 3 months was fixed in advance as the single primary endpoint; the later timepoints, TTP and progression-free survival, were secondary, and immune-related adverse events were the fourth endpoint of the association screen. Overall survival was not pre-specified: the clinical extract carried no vital status, so it was reconstructed afterwards from two further institutional sources and is reported as a secondary, exploratory endpoint (Supplementary Methods, Section S3). 

The 3-month status is a binary summary of the clinical course, not a time-to-event analysis. It groups complete response, partial response and stable disease as favourable and progression or death as unfavourable, for four reasons. First, stable disease lasting at least 6 months counts as clinical benefit in the consensus definitions of the Society for Immunotherapy of Cancer (SITC) [42], although the same definitions would classify stable disease at 3 months as primary resistance if progression followed before 6 months. Second, status at a fixed time is comparable between patients with different follow-up times. Third, death is captured: 25 of 76 deaths occurred without documented progression (Section 2.11), and a progression-only endpoint would censor them. Fourth, the SITC defines primary resistance to anti-PD-(L)1 therapy as progression within about 6 months of starting treatment [42,43,44], so unfavourable status at 3 months is an early subset of primary resistance and the 6-month status approximates the full window. Only the binary status was retained in the clinical extract, so response and stable disease could not be analysed separately (Section S3). Time to progression and progression or death (labelled progression-free survival in Table 2 and in the external cohort) were analysed as secondary endpoints.

### 5.3. Genotyping and Call Resolution

Exome data were generated for a previously reported study [39]. A germline IL7 polymorphism was separately tested against immune-related adverse events by TaqMan genotyping over the same recruitment period [45], without reference to HLA or KIR. Germline DNA was captured with the Twist Human Core Exome 2.0 kit and sequenced paired-end 2 × 150 to a mean depth above 30×; reads were trimmed with fastp v0.23.4 [46] and aligned to GRCh38 with BWA-MEM [47]. HLA class I and II and KIR alleles were called from the resulting BAM files with T1K v1.0.5 [48]; call confidence was characterised from T1K quality and read-abundance scores. To validate the calls, we retyped a random subset of 14 samples (12.6% of the 111 audited) with OptiType v1.5.0 [49] and arcasHLA v0.6.0 [50] from the MHC-region reads of the same BAM files. OptiType, built for exome reads, agreed at 91.7% of alleles and 83.3% of genotypes at the three class I genes it types; arcasHLA, applied outside its intended RNA-seq domain, agreed at 69.6% and 50.0%, pooled over the eight loci compared (three class I and five class II), and bounds the validator rather than the typing (Tables S2 and S3). 

Call resolution was audited before any association analysis. It covers all 111 samples from which an *HLA* call could be produced, before the clinical exclusions that define the analysis cohort, so its denominators exceed the 102 patients carried into the association models, which are a strict subset of the 111. Of 1155 patient-by-locus units across 12 genes, 94.0% returned a single candidate genotype and 99.1% resolved unambiguously at the first field, because competing candidates almost always belonged to the same allele group; 94.3% resolved at two fields, and 10 units (0.9%) could not be resolved at the first field. All *HLA* analyses were therefore conducted at first-field resolution, except DQB1*03:01, reported at two fields because the first-field group DQB1*03 aggregates alleles with different peptide-binding motifs; two-field resolution at *HLA-DQB1* was 92.7% (101 of 109 audited units).

*KIR* calls did not meet the same standard: 195 of 1025 gene-present patient-by-gene units (19.0%) were ambiguous and none resolved at the first field, so *KIR* analysis was restricted to gene presence and *KIR*-*HLA* ligand pairs (Tables S2).

### 5.4. Derived Variables

An allele group is the first field of the allele name, for example A*02 for A*02:01. An allele group was testable if at least 5 of the 102 genotyped patients carried it and at least 5 did not, giving 36 class I and 34 class II groups; the rule was applied once, fixing the exposure set and the permutation correction before any endpoint was analysed. Because the analysis set shrinks at each landmark as patients become unevaluable, 15 of the 448 tests fall below these thresholds in their own set (14 with fewer than 5 carriers and 1 with fewer than 5 non-carriers); all 15 were retained in the primary analysis and flagged, and a Benjamini–Hochberg refit on the remaining 433 is reported as a sensitivity analysis (Tables S10b and S12). Class I supertypes followed Sidney and colleagues [17]; HLA evolutionary divergence was the mean Grantham distance between the peptide-binding domains of the two alleles at a locus, a measure of amino-acid dissimilarity in which a higher value means more dissimilar alleles [9,10] (Table S4).

*KIR*-*HLA* ligand pairs combined *KIR* gene presence with the corresponding C1, C2 or Bw4 ligand group, using the gene content conventions established for southern European populations [30].

### 5.5. Statistical Analysis

Six hypothesis families were fixed in advance: class I allele groups, class II allele groups, class I supertypes, *HLA* evolutionary divergence, *KIR* gene presence and *KIR*-*HLA* ligand pairs, with zygosity examined alongside divergence. Binary outcomes were modelled by Firth penalised logistic regression [51,52]. Ordinary logistic regression fails when an exposure perfectly predicts the outcome, as B*14 and C*08 do at the 3-month landmark (no carrier had unfavourable status); the Firth penalty keeps the estimates finite and stable in small groups. The odds ratio (OR) is the ratio of the odds of unfavourable status in carriers to the odds in non-carriers, so a value above 1 means higher odds in carriers and a value below 1 lower odds. Confidence intervals are profile penalised-likelihood intervals, whose lower limit can lie close to zero when a group has no events and is then reported to four decimals. Time-to-event analysis used Kaplan–Meier estimation, log-rank tests, which compare whole survival curves, and Cox regression, which gives a hazard ratio (HR), the relative rate of progression or death over time, where a value above 1 means a faster rate in the exposed group; restricted mean survival time was used as a complement where the proportional-hazards assumption was in doubt [53,54].

The false discovery rate, which is the expected proportion of false positives among the results called significant, was controlled by the Benjamini–Hochberg procedure [55] within each hypothesis family at each endpoint. Each adjusted *p* value is reported as a *q* value, and the effective number of independent tests was estimated from the correlation between exposures, because correlated exposures count as fewer than their number [56]. A Westfall–Young single-step minimum-p permutation procedure [57], which estimates the family-wise error rate (the probability of at least one false positive in a set of tests) by shuffling the outcome labels while keeping the exposures unchanged, was then applied to the 46 class I exposures at the primary endpoint, with 10,000 permutations of the outcome label, and also to the six divergence exposures within each endpoint and pooled across the four endpoints, to the wider sets tested at the 12-month endpoint (40 class II and divergence exposures; all 112 exposures) and to all 448 exposure-by-endpoint combinations (2000 to 10,000 permutations according to level). Family-wise error rates are reported at this ladder of nesting levels with Monte Carlo intervals (Table S11). Further permutation analyses compared the leading estimates with the largest odds ratio expected by chance among exposures of similar carrier frequency (frequency-matched null maxima); E-values [58], which give the strength of unmeasured confounding needed to explain away an estimate, and leave-one-out and bootstrap analyses complete the sensitivity workup (Tables S16 and S22).

Analyses used R 4.4.3 with logistf, survival, survRM2, metafor and ggplot2, with the random seed fixed; no clinical covariate was imputed. *p* and *q* values are given to three decimals (*p* < 0.001 below that), odds ratios and hazard ratios with 95% confidence intervals to two decimals (four decimals for limits that would round to zero), and each estimate with its number of patients. All tests were two-sided: Fisher exact test for contingency tables, the penalised-likelihood ratio test for Firth odds ratios, the Wald test for Cox hazard ratios, the log-rank test for Kaplan–Meier comparisons and the Wilcoxon rank-sum test for age, pack-years and ancestry components.

In sensitivity analyses (Table S26), genetic ancestry was estimated from genome-wide genotypes imputed from the sequencing reads with STITCH [59]: PLINK2 [60] scored each patient against eight reference ancestry panels (three Native American, two continental, one African American and two European), and a principal component analysis of the eight scores, centred and not scaled, was computed in the 191 samples of the imputed dataset, which include samples from other projects. In the plane of the first two components, a patient was classified as outside the main cluster when the robust Mahalanobis distance (minimum covariance determinant, fitted on the 98 patients) exceeded the 99th percentile of the chi-squared distribution with 2 degrees of freedom, a rule fixed before the outcome was examined (Figure S6). Smoking status and pack-years came from a separate curated clinical database and were matched by patient identifier. Each was entered as a covariate in Firth models for the three class I allele groups at the primary endpoint; ancestry was entered as an indicator of position outside the main cluster or as the two standardised components and was also handled by restriction to the main cluster. Two pack-years values recorded only as a lower bound (above 50 and above 100) were entered at that value, and patients without data were excluded from the corresponding models without imputation. Missing smoking data were described by start year, and the A*02 association was compared between patients with and without recorded status with an interaction term (post hoc). These analyses lie outside the correction structure of the screen.

Throughout, within-family *q* values control the expected fraction of false discoveries inside each hypothesis family, whereas the permutation family-wise error rates estimate the probability of even one false positive across families of correlated exposures; the two scales answer different questions and are reported side by side.

### 5.6. Comparison with a Public Checkpoint-Treated Cohort

SU2C-MARK provides germline class I alleles called by POLYSOLVER [61] for 309 patients with exome data, drawn from a cohort of 393 patients with advanced NSCLC treated with checkpoint blockade [62]; 297 were evaluable for progression-free survival in the released file, with 232 events. We carried *HLA*-A*02 and *HLA*-B*14 carriage into this dataset under identical exposure definitions. The frozen protocol pre-specified progression-free survival (PFS) as distributed as the primary external endpoint, because the clinician-assigned 3-month status has no equivalent in this cohort, and a 90-day landmark as the confirmatory outcome: unfavourable if progression or death occurred by day 90 and favourable if follow-up extended beyond day 90, with patients censored by day 90 excluded. The landmark is derived from follow-up and is therefore an analogue of the 3-month status, not the same measurement. It was analysed with Firth penalised logistic regression and compared with the internal odds ratio by Cochran *q* (Table S20c).

Compatibility between cohorts was assessed with Cochran Q, which tests whether the effect is the same in both cohorts, and I^2^, the proportion of the variability between estimates that reflects true differences between cohorts rather than chance; pooled estimates came from a DerSimonian–Laird random-effects model in metafor [63]. The analysis protocol was written and hash-stamped after the external analytic set had been assembled and its marginal distributions inspected, and before any analysis of exposure against outcome in that cohort; it is deposited with the analysis code at Zenodo (doi:10.5281/zenodo.23180011).

### 5.7. Use of Generative Artificial Intelligence

Generative artificial intelligence was used during the preparation of this manuscript. Anthropic Claude (Sonnet and Opus model families, 2025 and 2026 releases) was used for language editing and for drafting and reviewing analysis code, accessed through the Biomni platform (Phylo). The authors reviewed and edited all output and take full responsibility for the content of this publication.

## Figures and Tables

**Table 1:** Baseline characteristics of the 99 genotyped patients with an assessable 3-month landmark. Values are *n* (%) unless otherwise stated; percentages use available data. *p* values from Fisher’s exact test (categorical variables; exact Freeman-Halton test for ECOG and PD-L1 category) and the Wilcoxon rank-sum test (age).

Characteristic	Favourable (*n* = 68)	Unfavourable (*n* = 31)	Total (*n* = 99)	*p*	Missing
Patients, *n*	68	31	99		0
Age, years, median (IQR)	66.4 (59.4 to 71.5)	64.7 (61.1 to 70.0)	65.9 (59.8 to 71.0)	0.818	0
ECOG performance status: 0	18 (27.7)	3 (10.0)	21 (22.1)	0.039	4
1	45 (69.2)	23 (76.7)	68 (71.6)		
2 or more	2 (3.1)	4 (13.3)	6 (6.3)		
Sex: Female	14 (21.9)	7 (22.6)	21 (22.1)	1.000	4
Male	50 (78.1)	24 (77.4)	74 (77.9)		
Histology: Adenocarcinoma	38 (59.4)	22 (71.0)	60 (63.2)	0.365	4
Non-adenocarcinoma	26 (40.6)	9 (29.0)	35 (36.8)		
PD-L1 tumour proportion score: High (50% or above)	15 (24.6)	5 (17.2)	20 (22.2)	0.652	9
Low (1 to 49%)	24 (39.3)	11 (37.9)	35 (38.9)		
Negative (below 1%)	22 (36.1)	13 (44.8)	35 (38.9)		
Treatment: Chemo-immunotherapy	50 (79.4)	19 (63.3)	69 (74.2)	0.129	6
ICI monotherapy	13 (20.6)	11 (36.7)	24 (25.8)		
Any immune-related adverse event: No	30 (50.0)	14 (53.8)	44 (51.2)	0.816	13
Yes	30 (50.0)	12 (46.2)	42 (48.8)		
HLA-A*02 carrier: No	54 (79.4)	15 (48.4)	69 (69.7)	0.004	0
Yes	14 (20.6)	16 (51.6)	30 (30.3)		
HLA-B*14 carrier: No	56 (82.4)	31 (100.0)	87 (87.9)	0.016	0
Yes	12 (17.6)	0 (0.0)	12 (12.1)		
HLA-C*08 carrier: No	58 (85.3)	31 (100.0)	89 (89.9)	0.028	0
Yes	10 (14.7)	0 (0.0)	10 (10.1)		

**Table 2:** The three class I allele groups with the strongest associations at the primary endpoint. Odds ratios (OR) are from Firth penalised logistic regression with profile penalised-likelihood intervals, in 99, 94 and 91 patients at 3, 6 and 12 months, 88 patients in the adjusted model, which includes treatment type, PD-L1 (50% or above), histology and sex, and 84 with ECOG performance status added. Hazard ratios are from Cox regression (98 patients; Wald *p* values), and the adjusted hazard ratios (87 patients) include treatment type, PD-L1 (50% or above), histology, sex and ECOG performance status; PFS is the composite of progression or death. B*14 and C*08 are in near-complete linkage disequilibrium (phi 0.903); the lower limits near zero are profile penalised-likelihood limits, as no carrier of either had an unfavourable 3-month status.

	HLA-A*02	HLA-B*14	HLA-C*08
Carriers, *n* (%)	30/99 (30.3%)	12/99 (12.1%)	10/99 (10.1%)
Unfavourable status at 3 months, OR (95% CI)	4.00 (1.64 to 10.07), *p* = 0.002	0.07 (0.0006 to 0.58), *p* = 0.008	0.09 (0.0007 to 0.73), *p* = 0.019
Adjusted for treatment, PD-L1 (50% or above), histology, sex, OR (95% CI)	5.00 (1.90 to 14.00), *p* = 0.001	0.08 (0.0006 to 0.62), *p* = 0.011	0.10 (0.0007 to 0.83), *p* = 0.029
Additionally adjusted for ECOG, OR (95% CI)	5.35 (1.90 to 16.82), *p* = 0.001	0.08 (0.0006 to 0.67), *p* = 0.014	0.10 (0.0007 to 0.86), *p* = 0.033
n for the ECOG-adjusted model	84	84	84
Unfavourable status at 6 months, OR (95% CI)	2.64 (1.09 to 6.61), *p* = 0.031	0.14 (0.01 to 0.62), *p* = 0.007	0.17 (0.02 to 0.80), *p* = 0.023
Unfavourable status at 12 months, OR (95% CI)	1.39 (0.55 to 3.72), *p* = 0.495	0.18 (0.04 to 0.63), *p* = 0.007	0.25 (0.06 to 0.96), *p* = 0.044
Median TTP, carriers vs. non-carriers, months	6.8 vs. 11.2	28.9 vs. 8.4	21.7 vs. 9.9
TTP hazard ratio (95% CI)	1.42 (0.81 to 2.49), *p* = 0.217	0.36 (0.14 to 0.90), *p* = 0.029	0.54 (0.21 to 1.35), *p* = 0.185
TTP hazard ratio adjusted (95% CI)	1.29 (0.68 to 2.46), *p* = 0.432	0.30 (0.12 to 0.78), *p* = 0.014	0.41 (0.16 to 1.07), *p* = 0.067
Schoenfeld residual *p*, TTP	0.129	0.158	0.068
Median PFS, carriers vs. non-carriers, months	3.2 vs. 8.3	21.7 vs. 6.5	20.8 vs. 7.3
PFS hazard ratio (95% CI)	1.29 (0.80 to 2.06), *p* = 0.297	0.37 (0.17 to 0.81), *p* = 0.013	0.48 (0.21 to 1.10), *p* = 0.081
PFS hazard ratio adjusted (95% CI)	1.28 (0.76 to 2.17), *p* = 0.354	0.30 (0.13 to 0.68), *p* = 0.004	0.35 (0.14 to 0.83), *p* = 0.017
Schoenfeld residual *p*, PFS	0.172	0.036	0.030
Permutation FWER within locus	0.018	0.057	0.174
Permutation FWER across the 36 class I allele groups	0.044	0.142	0.382

## Data Availability

The data supporting the findings of this study are not publicly available. Individual-level germline genotype data and linked clinical data cannot be deposited in an open repository because the consent obtained from participants does not cover open publication of individual genetic data. Aggregate carrier frequencies for all testable HLA class I and class II allele groups and all KIR genes, together with the complete association output for all six hypothesis families, are provided in the Supplementary Materials. Individual-level data are available from the corresponding author on reasonable request, subject to approval by the regional research ethics committee and to a data transfer agreement. The external comparison cohort is public: only the already published POLYSOLVER germline class I calls (sheet Table_S9_Polysolver_HLA_Results of the supplementary tables of the open-access report [62]) and the clinical annotations distributed with them were used. The file was downloaded on 14 August 2026 and its checksum verified; the SU2C-MARK data repository is available under a CC-BY 4.0 licence at Zenodo (doi:10.5281/zenodo.7625517) [62]. No controlled-access resource (dbGaP) and no raw sequencing data were accessed. The analysis code, the dated internal analysis protocol, the frozen pre-specification documents for the external validation and the rules used to audit the manuscript’s numbers are deposited at Zenodo (doi:10.5281/zenodo.23180011). The deposit contains no individual-level data.

## Supplementary Materials

The following supporting information can be downloaded at: https://www.mdpi.com/article/10.3390/ijms27219933/s1.
